# Pyogenic Liver Abscess Complicating Acute Cholecystitis: Different Management Options

**DOI:** 10.3390/medicina58060782

**Published:** 2022-06-09

**Authors:** Daniel Paramythiotis, Anestis Karakatsanis, Eleni Karlafti, Stella Bareka, Elizabeth Psoma, Adam A. Hatzidakis, Antonios Michalopoulos

**Affiliations:** 1 Department of Surgery, AHEPA General University Hospital, Aristotle University of Thessaloniki, 54124 Thessaloniki, Greece; danosprx@auth.gr (D.P.); ankarakatsanis@gmail.com (A.K.); amichal@auth.gr (A.M.); 2 Department of Internal Medicine, AHEPA General University Hospital, Aristotle University of Thessaloniki, 54636 Thessaloniki, Greece; linakarlafti@hotmail.com; 3 Emergency Department, AHEPA General University Hospital, Aristotle University of Thessaloniki, 54124 Thessaloniki, Greece; 4 Department of Radiology, AHEPA General University Hospital, Aristotle University of Thessaloniki, 54124 Thessaloniki, Greece; elizabethpsoma@gmail.com (E.P.); adamhatz@auth.gr (A.A.H.)

**Keywords:** pyogenic liver abscess, cholecystitis, percutaneous, cholecystectomy

## Abstract

Acute cholecystitis, which is usually associated with gallstones is one of the most common surgical causes of emergency hospital admission and may be further complicated by mural necrosis, perforation and abscess formation. Perforation of the gallbladder is a relatively uncommon complication of acute cholecystitis (0.8–3.2% in recent reviews). The intrahepatic perforation causing a liver abscess is an extremely rare condition, anecdotally reported in the scientific literature, even in the rare types of subacute or acute perforation. Liver abscess caused by gallbladder perforation can be a life-threatening complication with a reported mortality of 5.6%. The treatment of synchronous pyogenic liver abscess and acute cholecystitis may be challenging. We reported three cases of liver abscess due to acute cholecystitis in which different therapeutical approaches were employed. The first case was treated with antibiotics and interval laparoscopic cholecystectomy; the second case was treated with emergency cholecystectomy; and the third case with percutaneous aspiration of the abscess only. The appropriate therapeutical method in these cases depends on the patient’s clinical condition, the on-site expertise that is available in the hospital, and the experience of the surgeon.

## 1. Introduction

A pyogenic liver abscess (PLA) is a rather rare entity with an increasing level of occurrence in western countries ranging from 1.1/100,000 to 3.6/100,000 people, and a substantial mortality rate which is estimated to be between 5.6 and 10%. Mortality may even reach 22% when there are multiple PLAs [1,2]. The formation of a PLA is associated with cholelithiasis in as many as 15% of patients and with biliary disease in general in up to 21.9% of patients. Reviews of patients with acute cholecystitis report that the course of a substantial 4.8–15% of patients may be complicated by a synchronous liver abscess [1].

There are limited published cases pertaining to cholecystitis that is complicated by a synchronous liver abscess [3,4,5,6,7]. Appropriate treatment depends on the severity of cholecystitis [8] and could either involve cholecystectomy alone or in combination with percutaneous abscess drainage. The therapeutical approach also depends on the age and comorbidities of the patients. Acute cholecystitis is one of the most frequent conditions requiring abdominal surgery in emergencies in elderly people [9] The current guidelines recommend surgery as soon as possible because evidenced-based clinical studies have confirmed that early treatment reduces the total hospital stay and does not increase the complication or conversion rates [10,11,12,13,14]. The present contraindications for laparoscopic cholecystectomy are few and may be classified as absolute (uncorrected coagulopathy, high anesthetic and surgical risk, gallbladder carcinoma) or relative. The latter includes either general conditions (end-stage liver disease) or local findings (previous surgery in the upper abdominal region, calcified gallbladder, cholecysto-enteric fistula, Mirizzi’s syndrome) [15].

Previously published studies found that severe local inflammation, as well as a high Charlson Comorbidity Index (CCI) score and high values of total bilirubin could favor open surgery or conversion. Other unquantifiable factors, such as local anatomy, tissue friability, or the surgeon’s experience may play a significant role in the decision to convert to open surgery. There are concerns about using the laparoscopic approach in patients with respiratory and cardiovascular comorbidities due to the metabolic effects of the induced pneumoperitoneum. This loss of reserve capacity is the single most important factor that decreases the elderly patient’s ability to tolerate operations. The proper management of fluid and electrolyte replacement, respiratory management to prevent atelectasis and pneumonia, and monitoring for possible cardiac complications are necessary to minimize the risk of systemic complications in the perioperative period [16,17,18,19]. Consequently, patients over 50 years of age in the presence of cardiovascular comorbidities or diabetes should be closely monitored in the postoperative period to avoid cardiovascular ischemic incidents and cardiovascular decompensation [15].

Although the same observation applies in patients suffering from diabetes mellitus (DM), these patients tend to develop more severe complications, have longer hospital stays, and suffer higher fatality rates [20]. Some studies mention a higher degree of gallbladder distension and an increased wall tension secondary to kinetic disorders caused by microangiopathy and diabetic neuropathy. Metabolic disorders and DM-related gallstone formation may play a role that is not fully elucidated [21]. Chronically elevated blood sugar levels alter the immune response and render the diabetic more susceptible to infections by various mechanisms, such as glycosylation of the complement proteins, inhibition of immunoglobulin-mediated opsonization of bacteria, inhibition of neutrophil migration phagocytosis, and apoptosis [22,23,24]. Moreover, septic site infection and wound dehiscence were encountered to be more frequent in diabetic patients [25,26,27,28,29]. In this regard, the advantages of the laparoscopic approach are extremely important in preventing perioperative morbidity [21].

In this article, we report three cases of patients with cholecystitis and synchronous liver abscess where the therapeutical approaches were different. The first case was treated with antibiotics and interval laparoscopic cholecystectomy, the second case was treated with emergency cholecystectomy and the third case with percutaneous aspiration only.

## 2. Case Reports

### 2.1. Case 1

A 69-year-old Caucasian woman presented to the emergency department because of acute epigastric pain radiating to her back of a 10-h duration. The patient reported that she had a similar incident six months ago; however, no diagnosis for that episode was sought or provided. She had a history of type 2 diabetes mellitus and arterial hypertension. Her regular medications consisted of metformin 850 mg b.d. and olmesartan 40 mg o.d.

Her vital signs were within normal values except for the presence of hypertension (BP 180/101 mmHg), while the physical exam was significant only for tenderness in the epigastric region. Initial laboratory values revealed normal white blood cell levels, elevated liver enzymes SGOT 8.6667 μmol/(s•L), SGPT 6.2833 μmol/(s•L); a total bilirubin of 33.35 •mol/L, CRP 1820 nmol/L; and normal amylase levels.

The chest and abdominal X-rays were normal. An abdominal ultrasound exam was performed which revealed a distended gallbladder with multiple intraluminal stones, the biggest one measuring 2 cm. The diameter of the gallbladder was measured at 9.5 cm without the presence of pericholecystic fluid. The ultrasound also revealed intrahepatic ductal dilatation and a common bile duct of 10 mm in diameter, containing possible hyperechoic masses. 

The patient was admitted to the ward and began treatment with I.V antibiotics consisting of ciprofloxacin and metronidazole.

During hospitalization, the patient remained hemodynamically stable and the pain gradually subsided. Later on, after four days of hospitalization, a new ultrasound was performed that revealed a normal appearing liver with homogeneous texture and relatively elevated echogenicity, due to grade I fatty infiltration. The intrahepatic ducts, as well as the bile duct were not dilatated and the gallbladder appeared distended with a diameter of 5 cm, exhibiting wall thickening and intraluminal stones. The same day, the clinical status of the patient deteriorated. The patient became febrile (38 °C) with pain upon palpation of the right upper abdominal quadrant. Laboratory tests revealed the presence of obstructive jaundice and the levels of CRP raised to 2914.3 nmol/L. The antibiotic treatment was upgraded to piperacillin/tazobactam with the addition of metronidazole. 

**Figure 1:**
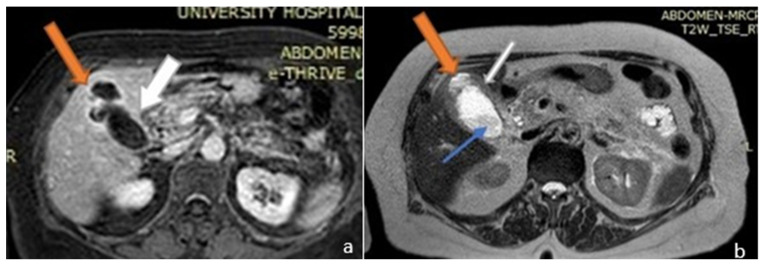
Magnetic resonance imaging (MRI) image of the upper abdomen. (**a**) Axial T1 post contrast and (**b**) T2. The gall bladder mucosa shows interrupted enhancement with a localized perforation anteriorly, close to the fundus. Orange arrows: liver abscess; white arrows: gallbladder; blue arrow: gallbladder stone.

**Figure 2:**
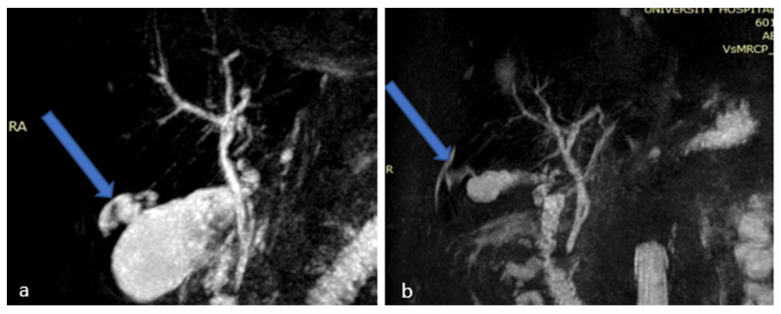
Magnetic Resonance Cholangiopancreatography (MRCP) images (**a**,**b**) showing gallbladder perforation (blue arrows).

On the patient’s seventh day of hospitalization, a magnetic resonance cholangiopancreatography (MRCP) with (IV) contrast injection was performed. This revealed an over-distended gallbladder with sludge and lithiasis, as well as mural thickening with contrast enhancement. In the non-dependent area of the gallbladder there was a T2w hyperintense region, demonstrating restricted diffusion. The exam also revealed a disruption of the gallbladder wall which displayed as a mural defect. In this area, a 3 cm collection with stratified signal abutting the gallbladder fundus and causing intrahepatic biliary dilatation was shown. There was bile leakage into the adjacent liver parenchyma due to perforation, resulting in the formation of an intrahepatic abscess (Figure 1 and Figure 2).

The patient initially opted for non-operative management, although the relevant risks were clearly communicated to her. She continued to be treated with piperacillin/tazobactam/metronidazole and her fever, as well as her pain, finally resolved on the 10th day. The inflammation markers improved within three days and the patient was discharged after 15 days of hospitalization. She was kept on oral ceftoral at 400 mg o.d. for four days and suggested a diet free of fat. A follow-up visit and a repeat ultrasound were scheduled 15 days later. 

On her follow-up visit, the patient was asymptomatic. A repeat ultrasound, performed 22 days after discharge, was only significant for a nondistended gallbladder, albeit with the presence of intraluminal stones and bile. 

Thirty-eight days later, the patient was readmitted to the hospital with a presumed diagnosis of cholangitis. Her vital signs were within normal range and the physical exam showed hyperactive bowel sounds and mild tenderness while palpating the right upper quadrant. The laboratory results were unremarkable except for an elevated CRP at 895.23 nmol/L.

**Figure 3:**
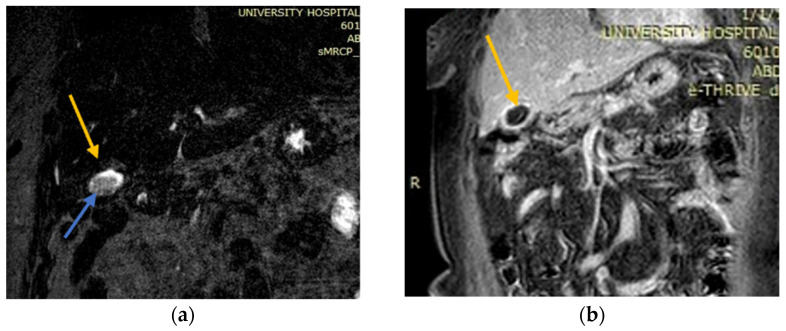
MRI images which showed findings consistent with acute cholecystitis. (**a**) Coronary MRI-image T1 post contrast of a gallbladder with diffuse mural thickening and contrast enhancement; (**b**) T2 MRI source image showing no perforation of the gallbladder. MRCP excluded choledocholithiasis and dilatation of the common bile duct. Yellow arrows: gallbladder; blue arrows: intraluminal stone and bile.

A new MRI was performed which showed an over-distended gallbladder with diffuse mural thickening and contrast enhancement, findings consistent with acute cholecystitis. No perforation of the gallbladder was shown, as well as no choledocholithiasis, nor dilatation of the common bile duct (Figure 3). 

The patient was again hospitalized for eight days and treated with piperacillin/tazobactam. Seven days after discharge, she underwent an elective laparoscopic cholecystectomy with an uneventful postoperative course. The patient was discharged on the first postoperative day with oral antibiotic treatment for two more weeks while a follow-up visit did not reveal any further complications.

### 2.2. Case 2

A 79 year-old Caucasian man presented to the emergency department due to acute pain during the last 24 h, originating from the right upper quadrant. The patient mentioned the presence of a mild, intermittent pain in the same area during the previous month. He also complained about anorexia, nausea, and fever during the last four days. 

On admission, he was febrile (39 °C) with otherwise normal vital signs. The clinical examination revealed tenderness in the right upper quadrant with a positive Murphy’s sign, while no palpable mass was appreciated. The pain was aggravated by respiratory movement and no other symptoms were reported. Laboratory studies were significant for leucocytosis (21.19 K/μL), an elevated CRP of 1333.3 nmol/L, γGT 4.56 μmol/(s•L) and total bilirubin at 25.65 •mol/L. 

**Figure 4:**
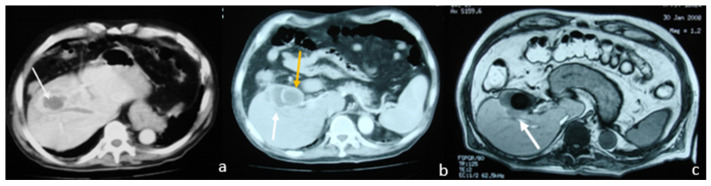
Contrast-enhanced Computed Tomography (CT) and MRI images which show overdistended and inflammatory gallbladder and a nodular lesion occupying the liver. (**a**) Contrast-enhanced CT: nonhomogeneous, hypodense mass located in the right lobe of the liver (white arrow); (**b**) contrast-enhanced CT: gallbladder with wall thickening and a large stone (yellow arrow) causing gallbladder neck obstruction (white arrow: liver abscess); (**c**) T1 out-of-phase axial MRI image: nodular lesion (white arrow) occupying the fourth and eighth liver segments in continuation with the overdistended and inflammatory gallbladder. It also revealed diffuse mural thickening with alternating layers of abnormal signal, as well as pericholecystic and perihepatic fluid.

An abdominal CT scan detected a nonhomogeneous, hypodense mass in the right lobe of the liver, while a small discontinuation of the thickened gallbladder wall was evident and in communication with the intrahepatic collection. The CT scan also revealed a large stone causing gallbladder neck obstruction (Figure 4a,b).

Magnetic resonance imaging (MRI) showed a nodular liver lesion with rim enhancement occupying the fourth and eighth segments in continuation with the over-distended and inflammatory gallbladder. It also revealed a large stone causing gallbladder neck obstruction, plus diffuse mural thickening with alternating layers of abnormal signal, as well as pericholecystic and perihepatic fluid. All these findings were consistent with the presence of a liver abscess (Figure 4c).

**Figure 5:**
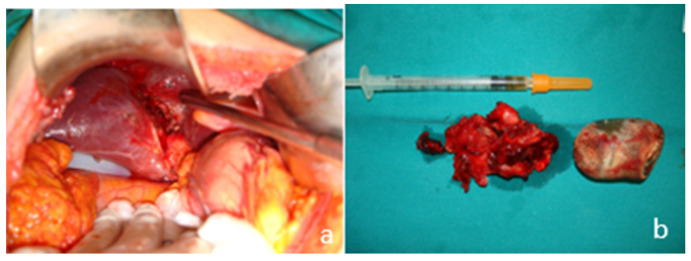
Intraoperative images. (**a**) Intrahepatic perforation of the gallbladder; (**b**) the resected gallbladder and the stone causing the neck obstruction.

The patient was admitted with the diagnosis of acute cholecystitis complicated by an intrahepatic abscess. He underwent emergency open cholecystectomy, due to the impacted large stone in the neck of the gallbladder (Figure 5). At the gallbladder’s fundus a fistulous communication between the gallbladder and the abscess cavity was identified, which developed in the subcapsular area of the right hepatic lobe on its visceral surface, containing a mix of bile and pus. The abscess cavity was washed out and the cholecystectomy performed. The abscess cavity and the subhepatic space were drained. The patient was further treated with I.V antibiotics (tetracycline, clavulanic acid and metronidazole) during hospitalization.

The patient’s postoperative course was uneventful and the drains were removed on the second (the intracavitary one) and third (subhepatic one) postoperative day. A follow-up CT scan showed that the abscess cavity had disappeared. He was discharged from the hospital five days after surgery on oral antibiotics (ciprofloxacin 500 mg b.d.) for 2 weeks and prescribed home wound care. A follow-up visit did not reveal any complications.

### 2.3. Case 3

A 54-year-old Caucasian man presented to the emergency department because of a 12-h acute pain that was localized in the right upper abdominal quadrant, and a fever of 39 °C. He was discharged one week prior from another hospital with the diagnosis of acute cholecystitis in remission complicated by a liver abscess. He had been receiving metronidazole PO for seven days.

The patient had no significant past medical history except for his previous hospitalization, a chronic alcohol consumption (four drinks per day), and a smoking history of 350 cigarettes/week. The patient had no known drug allergies and he reported no environmental, food, or seasonal allergies.

On arrival, a physical examination revealed pain in the right hypochondrium during deep palpation, with rebound tenderness, a positive Murphy’s sign without hepatosplenomegaly, and present bowel sounds. His vital signs were normal except for a temperature of 39.5 °C and his laboratory results were significant for leucocytosis (18.09 K/μL), abnormal ALP levels of 5.62 μmol/(s•L), and a raised CRP at 125.34 •mol/L. 

**Figure 6:**
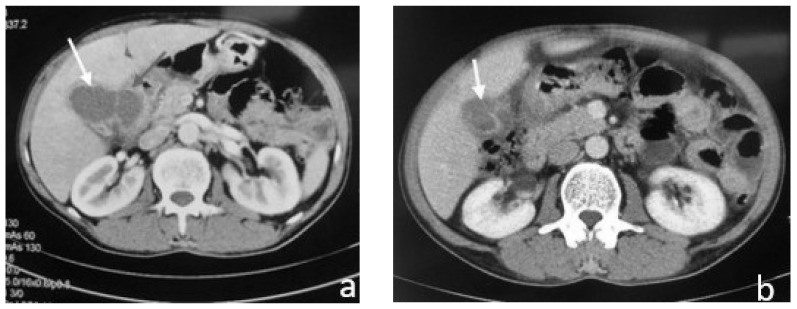
Contrast-enhanced CT shows: (**a**) gallbladder associated with pericholecystic fat stranding, gallbladder wall thickening, and liver abscess (white arrow); (**b**) gallbladder with wall thickening and a so-called “ring sign”, without central enhancement (white arrow).

An abdominal CT revealed an enlarged liver and a complex low-attenuation mass in the upper margin of the gallbladder. After contrast injection, this mass showed a peripheral enhancement forming a hyperdense border, the so-called “ring sign”, without central enhancement. This finding was consistent with an abscess. The gallbladder demonstrated uniform wall thickening (Figure 6).

**Figure 7:**
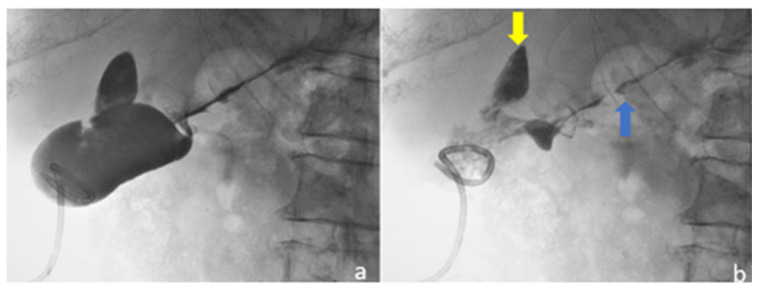
Ultrasound-guided percutaneous cholecystostomy with pigtail catheter inside gallbladder. (**a**) Gallbladder opacification through drainage catheter with occluded cystic duct; (**b**) after aspiration of the contrast medium, multiple small stones are seen in the gallbladder and contrast stain is also seen outside the gallbladder towards the liver parenchyma (yellow arrow) and the lower liver capsule (blue arrow).

The patient was admitted with the diagnosis of acute cholecystitis complicated by an intrahepatic abscess. He was immediately treated with I.V antibiotics (tetracycline, clavulanic acid and metronidazole) and an ultrasound-guided cholecystostomy was carried out, during which 100 mL of pus was aspirated (Figure 7). Cultures of pus grew *Klebsiella oxytoca* and *Escherichia coli*. The patient responded well to aspiration and antibiotic therapy and the inflammatory markers improved. He was discharged the next day on oral antibiotics for two weeks with a scheduled follow-up to arrange for interval cholecystectomy.

**Figure 8:**
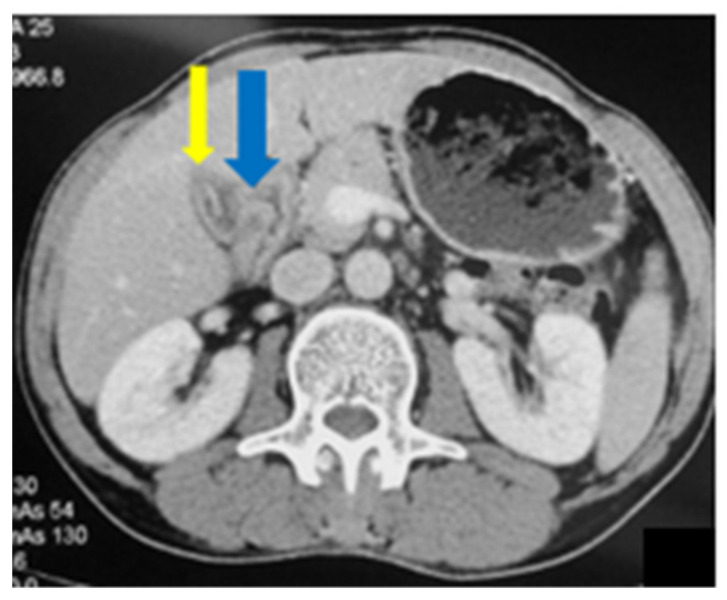
Abdominal CT: mild wall thickening of the gallbladder (blue arrow) with pericholecystic inflammatory changes (yellow arrow).

A follow-up CT scan was performed which revealed complete resolution of the liver abscess, as well as a mild wall thickening of the gallbladder with pericholecystic, inflammatory changes (Figure 8). The patient was booked for an elective laparoscopic cholecystectomy which he underwent two weeks later with no complications.

## 3. Discussion

Ten to twenty percent of the adult population is affected by cholelithiasis, while only 1–2% of people with cholelithiasis will suffer from acute cholecystitis during their lifetime. Gallbladder perforation (GBP) is a rare complication of acute cholecystitis that was first described by Duncan [30] in 1844 and that occurs in 0.8–3.2% of all such cases [30,31,32].

The pathophysiology of acute cholecystitis originates most commonly from the persistent occlusion of the cystic duct by an impacted stone (90–95%), an occlusion that causes biliary stasis, increased gallbladder wall tension and subsequently, epithelial injury, the release of phospholipases, the degradation of adjacent cell membranes and intense inflammatory reaction [33]. Life-threatening complications, such as wall necrosis and subsequent perforation may develop, either early in the acute cholecystitis phase or in the weeks after the onset of the disease; long-standing cholelithiasis, male gender, advanced age, arteriosclerosis, diabetes, immunosuppression, or steroid treatment are the most important risk factors. Older patients may also present with an atypical picture of acute cholecystitis, as pain is absent in 5–25% of this population and 30–50% of these patients may be afebrile [34,35]. Perforation occurs more often in the fundus of the gallbladder (33.3–42.9%) since this is the most distal part to the cystic artery and most prone to ischemic changes [36,37].

Gallbladder perforations are classified following Niemeir’s proposal in 1934, which is based on the direction of the perforation and the acuity of the underlying process [4]. In 1951 the above classification was modified by Fletcher and Radvein [38]. Briefly, type I perforation presents as an acute disease with perforation into the free abdominal cavity, whereas type II perforation is characterized as a subacute stage with development of a pericholecystic abscess, which may involve the liver parenchyma through direct spreading of the infection and form a pyogenic liver abscess (PLA). Finally, type III perforation arises in chronic cholecystitis with the development of bilio-enteric fistulae. It has been reported that type II is the most frequent kind of perforation [39]. A fourth type of perforation was added in 1987 by Anderson [40]. This new type was described as the development of a cholecysto-biliary fistula [40], however, this type is included in type III, and therefore is not recognized as a special type [7].

Several risk factors for PLAs, except for biliary disease have been identified, such as cirrhosis, diabetes mellitus, liver transplantation and malignancy. Diabetes mellitus has been estimated to complicate the clinical course of half of the patients that are treated for PLA and in need of treatment in an intensive care unit [1,41].

A useful distinction of pyogenic liver abscesses differentiates them as “pericholecystic”, that mainly involve liver segments IV or V, and “distant”, that may be located elsewhere in the liver parenchyma [1,41]. It has been postulated that the former may be caused by direct posterior GBP, especially in the event of an intrahepatic gallbladder, whereas the latter may be caused by hematogenic or biliary spread from acute cholecystitis, gallbladder empyema, or gallbladder gangrene. This apparently explains the different percentages of patients with acute cholecystitis and perforation (0.8–3.2%) vs. patients with acute cholecystitis and pyogenic liver abscess (4.8–15%).

The clinical presentation of patients suffering from acute cholecystitis complicated by perforation and abscess formation is often non-specific, in that both acute cholecystitis complicated by PLA formation and uncomplicated acute cholecystitis may present with a similar clinical picture and laboratory findings. Therefore, a diagnosis of synchronous PLA and acute cholecystitis may be difficult to establish based solely on clinical and laboratory data [42]. One distinguishing feature of this complication is the duration of pain, which may be present as long as 3–15 days before the admission of the patient to the hospital [42,43]. Another distinguishing feature is a reported sudden decrease in perceived pain, due to the relief of high intracystic pressure after the perforation [42]. It has been reported that Murphy’s sign might be absent, especially in patients with free abdominal fluid, such as patients on peritoneal dialysis, or when due to the covered perforation into the liver, the peritoneal layers may not be affected [44].

Imaging may help to distinguish between acute cholecystitis complicated by PLA and uncomplicated acute cholecystitis. Ultrasound is usually the first imaging method that is employed for patients suspected of having acute cholecystitis and it can be useful for evaluating a possible GBP. Detecting a defect in the gallbladder wall (“the hole sign”) is the only reliable sign of gallbladder perforation (55–70% sensitivity) [1,44], while other ultrasound findings, including pericholecystic effusion (type I), intracystic gas echo (type III), a partially obscured wall and a pericholecystic mass (type II), always depend on the type of perforation [1,39]. However, its accuracy is severely impaired by abundant bowel gas, lack of patient cooperation, and obesity. Contrast-enhanced ultrasound (CEUS) has been used to overcome these technical problems, and apart from clearly defining the defect of a hyper-enhanced wall it may more accurately depict a PLA, which during the arterial phase has a honeycomb-like appearance, heterogeneous enhancement, multiple septa and a few areas of non-enhancement. Furthermore, the use of CEUS may be helpful in determining the benign nature of the pericholecystic mass. Finally, the infused contrast requires no allergy test and causes no harm to liver or kidney function [39]. However, more studies must be performed before this technique is applied more frequently.

CT scans have the advantage of a better representation of extensive findings because of the bigger field of view, and they may demonstrate the extension of a lesion more clearly. CT may be extremely useful, especially in cases of discrepancies between clinical symptoms and an inconclusive ultrasound. It may also be used to evaluate for possible complications of acute cholecystitis and to better plan the possible surgery. However, regarding the detection of GBP, CT’s sensitivity is only a little better than ultrasound’s (80% sensitivity) [44]. In order to increase the accuracy of CT, it has been proposed that CT should be employed in the evaluation of any patient with acute cholecystitis who is >55 years old, has a temperature >38 °C, a WBC count >12,000/mL, AST > 50 IU/L and/or ALT > 75 IU/L, in order to identify PLAs [1]. 

A CT scan, however, involves exposure to ionizing radiation and the administration of intravenous contrast, which may cause acute kidney injury to volume-depleted patients. Furthermore, CT is unreliable for identifying gallstones as it underestimates gallbladder wall thickening and cannot detect a positive Murphy’s sign. Therefore, MRI may be employed as it allows for the accurate preoperative diagnosis of complications of cholecystitis, such as intramural necrosis, perforation and the presence of PLA. The drawbacks of this method include the fact that it is contraindicated in patients with cardiac pacemakers or implantable devices, and that its usefulness is limited in uncooperative or severely ill patients [34].

Various options exist for the treatment of patients who suffer from synchronous pyogenic liver abscess and acute cholecystitis. Nowadays, the recommended approach to PLA is mostly nonsurgical and relatively straightforward (e.g., antibiotics for small, multiple PLAs; needle aspiration or intrahepatic catheter placement for larger PLAs; and surgery for abscesses >5 cm or unstable patients, or in the event of the failure of conservative treatment) [1]. Despite very few reports of PLAs complicating acute cholecystitis, there is no consensus regarding the standard treatment of such conditions. 

Some authors managed such conditions with an open approach with an en-bloc resection of the abscess, the gallbladder and the gallbladder bed, as an atypical resection of liver segment V [32]. Others utilized an open or laparoscopic approach, depending on local conditions (intrahepatic gallbladder, ill-defined anatomy, high risk of damage to hilar structures), with cholecystectomy and deroofing of the abscess cavity [32,42]. In especially frail patients, conservative management with only percutaneous cholecystostomy and antibiotics have been successfully implemented [45], as well as US-guided percutaneous drainage of the collection ± cholecystostomy, followed by elective cholecystectomy further in the future [40,46]. Other authors recommend antibiotics and percutaneous cholecystostomy for source control (reducing the bacterial load without drainage of the PLA), followed by laparoscopic cholecystectomy 2–4 months after the initial management of the patient [1].

## 4. Conclusions

Gallbladder perforations that lead to liver abscesses are a rare complication of acute and chronic disease of the gallbladder, while intrahepatic perforations are even rarer. The appropriate therapeutic method in these cases depends on the patient’s clinical condition, the on-site expertise that is available at the hospital, and the experience of the surgeon. In our institution, all three approaches resulted in curing the patients.

## Data Availability

The data presented in this study is available from the corresponding author on demand.
